# Supplementary material for: LMIC-PRIEST: Derivation and validation of a clinical severity score for acutely ill adults with suspected COVID-19 in a middle-income setting
Source: PLoS One. 2023 Jun 14;18(6):e0287091. doi: 10.1371/journal.pone.0287091 (PMC10266677; doi:10.1371/journal.pone.0287091)
Supplement: S1 File — (DOCX) [file pone.0287091.s001.docx]

S1 Table: Categorisation of continuous variables using TEWS

| **Score** | **3** | **2** | **1** | **0** | **1** | **2** | **3** |
| --- | --- | --- | --- | --- | --- | --- | --- |
| **Respiratory Rate** |  | <9 |  | 9-14 | 15-20 | 21-29 | >29 |
| **Pulse Rate** |  | <41 | 41-50 | 51-100 | 101-110 | 111-129 | >129 |
| **Systolic BP** | ≤70 | 71-80 | 81-100 | 101-199 |  | >199 |  |
| **Temperature** |  | <35 |  | 35-38.4 |  | ≥38.5 |  |
| **Neuro** |  | Confused |  | Alert | Reacts to Voice | Reacts to pain | Unresponsive |

S2 Figure: Derivation of UK PRIEST Validation cohort

Initial PRIEST data set

*N = 22,445*

Age < 16

*N = 1,530*

Suspected or confirmed COVID patient (16 or over)

*N = 20,915*

Patient incomplete demographic data (Sex or Age) or outcome data

*N* = 217

Final Cohort

*N = 20,698*

S3 Table: Population characteristics Omicron period validation cohort

| **Characteristic** | **Statistic/level** | **Adverse outcome** | **No adverse outcome** | **Total** |
| --- | --- | --- | --- | --- |
|  | N | 2,787 (2%) | 137,733 (98%) | 140,520 |
| Age (years) | Mean (SD) | 48.8 (18.8) | 43.2 (17.1) | 43.3 (17.2) |
|  | Median (IQR) | 46 (33, 64) | 40 (29, 56) | 41 (29, 56) |
|  | Range | 16 to 95 | 16 to 110 | 16 to 110 |
| Sex | Male | 1,658 (59.5%) | 70,056 (50.9%) | 71,714 (51%) |
|  | Female | 1,129 (40.5%) | 67,677 (49.1%) | 68,806 (49%) |
| Comorbidities | Asthma/COPD | 474 (17%) | 20,755 (15.1%) | 21,229 (15.1%) |
|  | Other Chronic respiratory disease | 7 (0.3%) | 297 (0.2%) | 304 (0.2%) |
|  | Diabetes | 801 (28.7%) | 21,307 (15.5%) | 22,108 (15.7%) |
|  | Hypertension | 1,008 (36.2%) | 36,227 (26.3%) | 37,235 (26.5%) |
|  | Immunosuppression (HIV) | 488 (17.5%) | 24,430 (17.7%) | 24,918 (17.7%) |
|  | Heart Disease | 912 (32.7%) | 24,078 (17.5%) | 24,990 (17.8%) |
|  | Pregnant | 43 (1.5%) | 727 (0.5%) | 770 (0.6%) |
| AVPU | Missing |  |  | 3,249 (2.3%) |
|  | Alert | 1,702 (61.1%) | 125,337 (91%) | 127,039 (90.4%) |
|  | Voice | 96 (3.4%) | 1,916 (1.4%) | 2,012 (1.4%) |
|  | Confused | 180 (6.5%) | 5,327 (3.9%) | 5,057 (3.9%) |
|  | Pain | 201 (7.2%) | 975 (0.7%) | 1,176 (0.8%) |
|  | Unresponsive | 554 (19.9%) | 983 (0.7%) | 1,537 (2.3%) |
| Systolic BP (mmHg) | Missing |  |  | 3,707 (2.6%) |
|  | N | 2,688 | 134,125 | 136,813 |
|  | Mean (SD) | 126 (30) | 129.7 (25.3) | 129.6 (25.4) |
|  | Median (IQR) | 122 (107,143) | 127 (113,143) | 127 (113,143) |
|  | Range | 52 to 288 | 50 to 300 | 50 to 300 |
| Pulse rate (beats/min) | Missing |  |  | 3,582 (2.6%) |
|  | N | 2,694 | 134,244 | 136,938 |
|  | Mean (SD) | 100 (24.7) | 93 (21.1) | 93.1 (21.2) |
|  | Median (IQR) | 99 (83,115) | 92 (78, 106) | 92 (78,106) |
|  | Range | 12 to 300 | 10 to 300 | 10 to 300 |
| Respiratory rate (breaths/min) | Missing |  |  | 3,571 (2.5%) |
|  | N | 2,690 | 134,259 | 136,949 |
|  | Mean (SD) | 19 (5.5) | 18.1 (3.6) | 18.1 (3.6) |
|  | Median (IQR) | 19 (17,22) | 18 (16,20) | 18 (16,20) |
|  | Range | 8 to 60 | 1 to 60 | 1 to 60 |
| Oxygen saturation | Missing |  |  | 8,260 (5.9%) |
|  | N | 2,641 | 129,619 | 132,260 |
|  | Mean (SD) | 93.9 (8.1) | 96.3 (4.9) | 96.2 (5) |
|  | Median (IQR) | 96 (93, 99) | 97 (96, 99) | 97 (95, 99) |
|  | Range | 13 to 100 | 10 to 100 | 10 to 100 |
| Oxygen administration | Missing |  |  | 7,910 (5.6%) |
|  | N | 2,662 | 129,948 | 132,610 |
|  | 1 (air) | 1,516 (57%) | 123,044 (94.7%) | 124,560 (93.9%) |
|  | 2 (40% O2) | 58 (2.2%) | 2,407 (1.9%) | 2,465 (1.9%) |
|  | 3 (28% O2) | 2 (0.1%) | 82 (0.1%) | 84 (0.1%) |
|  | 4 (Nasal prongs) | 1,19 (4.5%) | 2,610 (2%) | 2,729 (2.1%) |
|  | 5 (FM neb) | 11 (0.4%) | 378 (0.3%) | 389 (0.3%) |
|  | 6 (rebreather mask) | 1,10 (4.1%) | 1,315 (1%) | 1,425 (1.1%) |
|  | 7 (nasal prongs and rebreather mask) | 17 (0.6%) | 112 (0.1%) | 1,29 (0.1%) |
|  | 8 intubated | 776 (29.2%) | 0 | 776 (0.6%) |
|  | 9 NIV | 53 (2%) | 0 | 53 (0.04%) |
| Temperature (°C) | Missing |  |  | 3,258 (2.3%) |
|  | N | 2,733 | 134,529 | 137,262 |
|  | Mean (SD) | 36.3 (1.1) | 36.3 (0.7) | 36.3 (0.7) |
|  | Median (IQR) | 36.3 (35.9, 36.7) | 36.3 (36, 36.6) | 36.3 (36, 36.6) |
|  | Range | 25 to 40 | 25 to 41.9 | 25 to 41.9 |
| Cough | Missing |  |  | 93,962 (30.8%) |
|  | Present | 80 (2.9%) | 3,500 (2.5%) | 3,580 (2.6%) |
| Fever | Missing |  |  | 41,524 (29.6%) |
|  | Present | 25 (0.9%) | 1,169 (0.9%) | 1,194 (0.9%) |
| COVID PCR | Positive | 2,119 (76%) | 26,485 (19.2%) | 28,604 (20.4%) |
| Hospital admission | ICU | 6,77 (24.3%) | 0 | 6,77 (0.5%) |
| Death | Within 30 days contact | 1,431 (51.4%) | 0 | 1,431 (1%) |

S4 Table: Population characteristics UK PRIEST validation cohort

| **Characteristic** | **Statistic/level** | **Adverse outcome** | **No adverse outcome** | **Total** |
| --- | --- | --- | --- | --- |
|  | N | 4,579 (22.1%) | 16,119 (77.9%) | 20,698 |
| Age (years)* | 16-49 | 369 (8.1%) | 5,256 (32.6%) | 5,625 (27.2%) |
|  | 50-65 | 981 (21.4%) | 4,186 (26%) | 5,167 (25%) |
|  | 66-80 | 1,527 (33.4%) | 3,727 (23.1%) | 5,254 (25.4%) |
|  | >80 | 1,702 (37.2%) | 2,950 (18.3%) | 4,652 (22.5%) |
| Sex | Male | 2,661 (58.1%) | 7,540 (46.8%) | 10,201 (49.3%) |
|  | Female | 1,918 (41.9%) | 8,579 (53.2%) | 10,497 (50.7%) |
| Comorbidities | Asthma | 556 (12.1%) | 2,820 (17.5%) | 3,376 (16.3%) |
|  | Other Chronic respiratory disease | 1,045 (22.8%) | 2,693 (16.7%) | 3,738 (18.1%) |
|  | Diabetes | 1,274 (27.8%) | 2,816 (17.5%) | 4,090 (19.8%) |
|  | Hypertension | 1,828 (39.9%) | 4,538 (28.2%) | 6,366 (30.8%) |
|  | Immunosuppression | 171 (3.7%) | 456 (2.8%) | 627 (3%) |
|  | Heart Disease | 912 (32.7%) | 24,078 (17.5%) | 4,661 (22.5%) |
|  | Pregnant | 6 (0.1%) | 79 (0.5%) | 85 (0.4%) |
| AVPU | Missing |  |  | 2,063 (10%) |
|  | Alert | 3,030 (66.2%) | 13,335 (82.9%) | 16,385 (79.2%) |
|  | Voice | 263 (5.7%) | 234 (1.5%) | 497 (2.4%) |
|  | Confused | 557 (12.2%) | 907 (5.6%) | 1,464 (7.1) |
|  | Pain | 114 (2.5%) | 65 (0.4%) | 179 (0.9%) |
|  | Unresponsive | 77 (1.7%) | 33 (0.2%) | 1,10 (0.6%) |
| Systolic BP (mmHg) | Missing |  |  | 585 (2.8%) |
|  | N | 4,453 | 15,660 | 20,113 |
|  | Mean (SD) | 130.1 (26.7) | 135.9 (24.2) | 134.6 (24.9) |
|  | Median (IQR) | 129 (112,147) | 134 (120, 150) | 133 (118, 149) |
|  | Range | 47 to 254 | 37 to 264 | 37 to 264 |
| Pulse rate (beats/min) | Missing |  |  | 426 (2.1%) |
|  | N | 4,485 | 15,787 | 20,272 |
|  | Mean (SD) | 98.3 (23.3) | 93.9 (20.9) | 94.9 (21.5) |
|  | Median (IQR) | 97 (83,112) | 92 (80, 107) | 93 (80, 108) |
|  | Range | 8 to 209 | 11 to 220 | 11 to 220 |
| Respiratory rate (breaths/min) | Missing |  |  | 536 (2.6%) |
|  | N | 4,468 | 15,694 | 20,162 |
|  | Mean (SD) | 27.1 (8.5) | 22.2 (6.1) | 23.3 (7) |
|  | Median (IQR) | 25 (21,32) | 20 (18,24) | 22 (18,26) |
|  | Range | 6 to 99 | 5 to 99 | 5 to 99 |
| Oxygen saturation | Missing |  |  | 254 (1.2%) |
|  | N | 4,520 | 15,924 | 20,444 |
|  | Mean (SD) | 91.5 (8.8) | 95.6 (5.8) | 94.7 (6.8) |
|  | Median (IQR) | 84 (89, 96) | 97 (95, 98) | 96 (94, 98) |
|  | Range | 22 to 100 | 13 to 100 | 13 to 100 |
| Oxygen administration | On Oxygen | 1,964 (42.9%) | 2,249 (14%) | 4,213 (20.4%) |
| Temperature (°C) | Missing |  |  | 651 (2.3%) |
|  | N | 4,409 | 15,638 | 20,047 |
|  | Mean (SD) | 37.3 (1.2) | 37.1 (1) | 37.1 (1.1) |
|  | Median (IQR) | 37.2 (36.5, 38.2) | 36.9 (36.4, 37.7) | 37 (36.4, 37.8) |
|  | Range | 31.3 to 41.3 | 25.9 to 42.1 | 25.9 to 42.1 |
| Cough | Present | 2,659 (58.1%) | 10,211 (63.4%) | 12,870 (62.2%) |
| Fever | Present | 2,271 (49.6%) | 7,916 (49.1%) | 10,187 (49.2%) |
| Clinical impression | COVID | 3,419 (77.9%) | 10,518 (68.5%) | 13,937 (70.6%) |
| Organ Support | Any | 2,046 (44.7%) | 0 | 2,046 (9.9%) |
| Death | Within 30 days contact | 3,222 (70.4%) | 0 | 3,222 (15.6%) |

*Due to the small number of patients with some individual ages, age was categorised before receipt of the data to ensure anonymity

S5 Table: Restricted multivariable analysis complete case analysis (N=102, 402)

| **Lasso variable selection (restricted to 10) (Continuous variables modelled using fractional polynomials)** | | |
| --- | --- | --- |
| C-statistic: 0.867 (95%CI 0.861 to 0.873) | | |
| CITL:-0.016 (95%CI -0.054 to 0.022) | | |
| **Parameter** | **Coefficient** | |
|  | **Unstandardised** | **Standardised** |
| Age | 0.310 | 0.532 |
| No Supplemental Oxygen | -1.421 | -0.376 |
| (Saturation/10) ^3 -897.3 | -0.003 | -0.366 |
| No Diabetes | -0.464 | -0.174 |
| (Temperature/10) ^3 -48.11 | 0.041 | 0.128 |
| (AVCPU+1) ^3-1.4* | 0.01 | 0.104 |
| No Heart Disease | -0.225 | -0.085 |
| ln(respiratory rate/10) -0.60 | 0.448 | 0.085 |
| Systolic Blood Pressure | -0.002 | -0.051 |
| Heart Rate | 0.002 | 0.038 |
| Constant | -3.48 | -4.14 |
| **Lasso variable selection (restricted to 10) (Continuous variables (Continuous variables modelled using TEWS categories)** | | |
| C-statistic: 0.859 (95%CI: 0.854 to 0.863) | | |
| CITL: 0.126 (95% CI: 0.098 to 0.155) | | |
| **Parameter** | **Coefficient** | |
|  | **Unstandardised** | **Standardised** |
| Age | 0.031 | 0.525 |
| No Supplemental Oxygen | -1.426 | -0.377 |
| Saturation (point increase TEWS) | 0.473 | 0.422 |
| No Diabetes | -0.496 | -0.186 |
| AVCPU (point increase TEWS) | 0.266 | 0.121 |
| No Heart Disease | -0.228 | -0.086 |
| Heart Rate (point increase TEWS) | 0.094 | 0.086 |
| Respiratory Rate (point increase TEWS) | 0.141 | 0.081 |
| Male | 0.035 | 0.017 |
| No Hypertension | 0.021 | 0.009 |
| Constant | -4.037 | -4.179 |

S6 Table: Restricted Deterministic imputation (N=152, 782)

| **Lasso variable selection (restricted to 10) (Continuous variables modelled using TEWS categories)** | | |
| --- | --- | --- |
| C-statistic: 0.850 (95%CI 0.845 to 0.855) | | |
| CITL: -0.018 (95% CI -0.045 to 0.009) | | |
| **Parameter** | **Coefficient** | |
|  | **Unstandardised** | **Standardised** |
| Age | 0.024 | 0.41 |
| No Supplemental Oxygen | -1.676 | -0.469 |
| Saturation (point increase TEWS) | 0.372 | 0.35 |
| No Diabetes | -0.52 | -0.203 |
| AVCPU (point increase TEWS) | 0.518 | 0.281 |
| No Heart Disease | -0.019 | -0.007 |
| Heart Rate (point increase TEWS) | 0.024 | 0.022 |
| Respiratory Rate (point increase TEWS) | 0.073 | 0.046 |
| Male | 0.043 | 0.022 |
| Temperature (point increase TEWS) | 0.024 | 0.014 |
| Constant | -3.188 | -3.748 |

S7 Table: Restricted multivariable analysis, using multiple imputation (10 imputations; N=152,782 each imputation)

| **Lasso variable selection (restricted to 10) (Continuous variables modelled using fractional polynomials)** | | |  |
| --- | --- | --- | --- |
| Mean C-statistic: 0.869 (95%CI 0.865 to 0.873) | | |  |
| Mean CITL: -0.017 (95%CI -0.042 to 0.008) | | |  |
| **Parameter** | **Mean coefficient** | | **Times selected (*n*)** |
|  | **Unstandardised** | **Standardised** | 10 |
| (Age/10) ^2 | 0.024 | 0.419 | 10 |
| No Supplemental Oxygen | -1.667 | -0.480 | 10 |
| (Saturation/10) ^3 | -0.003 | -0.342 | 10 |
| No Diabetes | -0.486 | -0.190 | 10 |
| (Temperature/10) ^3 | 0.023 | 0.077 | 10 |
| No Heart Disease | -0.006 | -0.005 | 10 |
| (AVCPU+1) ^3 | 0.017 | 0.274 | 10 |
| (Systolic Blood Pressure/100) ^3 | -0.0002 | -0.006 | 6 |
| ln(respiratory rate/10) | 0.494 | 0.099 | 10 |
| Heart Rate | 0.001 | 0.031 | 10 |
| Male | 0.025 | 0.015 | 10 |
| Cough | 0.006 | 0.001 | 1 |
| Constant | -2.056 | -3.796 |  |
| **Lasso variable selection (restricted to 10) (Continuous variables modelled using TEWS Categories)** | | |  |
| C-statistic: 0.864 (95% CI 0.86 to 0.868) | | |  |
| CITL:-0.02 (95% CI -0.047 to 0.008) | | |  |
| **Parameter** | **Coefficient** | | **Times Selected (*n*)** |
|  | **Unstandardised** | **Standardised** |  |
| Age | 0.022 | 0.391 | 10 |
| No Supplemental Oxygen | -1.426 | -0.486 | 10 |
| Saturation (point increase TEWS) | 0.402 | 0.387 | 10 |
| No Diabetes | -0.501 | -0.195 | 10 |
| AVCPU (point increase TEWS) | 0.51 | 0.281 | 10 |
| No Heart Disease | -0.02 | -0.008 | 10 |
| Heart Rate (point increase TEWS) | 0.035 | 0.032 | 10 |
| Respiratory Rate (point increase TEWS) | 0.175 | 0.106 | 10 |
| Male | 0.041 | 0.021 | 10 |
| Temperature (point increase TEWS) | 0.048 | 0.027 | 10 |
| Cough | 0.004 | 0.001 | 1 |
| Constant | -3.382 | -3.186 |  |

S8 Table: Multivariable Analysis complete case analysis (N=102, 402)

| **Lasso variable selection (unrestricted) (Continuous variables modelled using fractional polynomials)** | | |
| --- | --- | --- |
| C-statistic: 0.868 | | |
| CITL:-0.014 | | |
| **Parameter** | **Coefficient** | |
|  | **Unstandardised** | **Standardised** |
| Age | 0.036 | 0.617 |
| No Supplemental Oxygen | -1.433 | -0.379 |
| (Saturation/10) ^3 -897.3 | -0.003 | -0.369 |
| No Diabetes | -0.559 | -0.121 |
| (Temperature/10) ^3 -48.11 | 0.051 | 0.159 |
| No Heart Disease | -0.318 | -0.121 |
| (AVCPU+1) ^3-1.4* | 0.011 | 0.119 |
| Systolic Blood Pressure | -0.098 | -0.004 |
| ln(respiratory rate/10) -0.60 | 0.502 | 0.095 |
| Heart Rate | 0.004 | 0.081 |
| Male | 0.133 | 0.066 |
| No Hypertension | 0.126 | 0.056 |
| Not Pregnant | -0.546 | -0.046 |
| No Immunosuppression (HIV) | -0.103 | -0.039 |
| No Cough | 0.128 | 0.026 |
| No Other chronic lung disease | -0.191 | -0.008 |
| No Fever | -0.043 | -0.005 |
| Constant | -2.993 | -4.248 |
|  |  |  |
| **Lasso variable selection (unrestricted) (Continuous variables modelled using TEWS categories)** | | |
| C-statistic: 0.863 | | |
| CITL:-0.045 | | |
| **Parameter** | **Coefficient** | |
|  | **Unstandardised** | **Standardised** |
| Age | 0.034 | 0.591 |
| No Supplemental Oxygen | -1.433 | -0.393 |
| Saturation (point increase TEWS) | 0.474- | 0.424 |
| No Diabetes | -0.638 | -0.241 |
| AVCPU (point increase TEWS) | 0.294 | 0.133 |
| Respiratory Rate (point increase TEWS) | 0.168 | 0.097 |
| No Hypertension | 0.184 | 0.082 |
| Heart Rate (point increase TEWS) | 0.089 | 0.081 |
| No Heart Disease | -0.196 | -0.074 |
| Not Pregnant | -0.527 | -0.045 |
| Male | 0.08 | 0.04 |
| No Immunosuppression (HIV) | -0.075 | -0.028 |
| No Cough | 0.124 | 0.025 |
| Temperature (point increase TEWS) | 0.043 | 0.024 |
| No Other chronic lung disease | -0.367 | -0.015 |
| Systolic Blood Pressure (point increase TEWS) | -0.029 | -0.012 |
| No Fever | 0.059 | 0.007 |
| Constant | -3.518 | -4.229 |

*AVCPU coded alert=0, Voice=1, Confusion=2, Pain= 3, Unresponsive =4

S9 Table: Unrestricted Deterministic Imputation (N=152,782)

| **Lasso variable selection (unrestricted) (Continuous variables modelled using TEWS categories)** | | |
| --- | --- | --- |
| C-statistic: 0.850 | | |
| CITL=-0.018 | | |
| **Parameter** | **Coefficient** | |
|  | **Unstandardised** | **Standardised** |
| Age | 0.0027 | 0.467 |
| No Supplemental Oxygen | -1.696 | -0.475 |
| Saturation (point increase TEWS) | 0.380 | 0.357 |
| No Diabetes | -0.638 | -0.232 |
| AVCPU (point increase TEWS) | 0.294 | 0.297 |
| Respiratory Rate (point increase TEWS) | 0.168 | 0.058 |
| No Hypertension | 0.184 | 0.066 |
| Heart Rate (point increase TEWS) | 0.089 | 0.062 |
| No Heart Disease | -0.196 | -0.041 |
| Not Pregnant | -0.527 | -0.037 |
| Male | 0.08 | 0.072 |
| No Immunosuppression (HIV) | -0.075 | 0.019 |
| No Cough | 0.124 | 0.034 |
| Temperature (point increase TEWS) | 0.043 | 0.044 |
| No Other chronic lung disease | -0.367 | -0.019 |
| Systolic Blood Pressure (point increase TEWS) | -0.029 | -0.016 |
| No Fever | 0.059 | 0.002 |
| Constant | -2.893 | -3.82 |

S10 Table: Multivariable analysis, using multiple imputation (10 imputations; N=152,782)

| **Lasso variable selection (unrestricted) (Continuous variables modelled using fractional polynomials)** | | |  |
| --- | --- | --- | --- |
| C-statistic: 0.87 | | |  |
| CITL: -0.15 | | |  |
| **Parameter** | **Average coefficient** | | **Number times selected** |
|  | **Unstandardised** | **Standardised** |  |
| (Age/10) ^2 | 0.027 | 0.463 | 10 |
| No Supplemental Oxygen | -1.691 | -0.486 | 10 |
| (Saturation/10) ^3 | -0.003 | -0.348 | 10 |
| No Diabetes | -0.5778 | -0.225 | 10 |
| (Temperature/10) ^3 | 0.034 | 0.114 | 10 |
| No Heart Disease | -0.123 | -0.048 | 10 |
| (AVCPU+1) ^3 | 0.019 | 0.295 | 10 |
| (Systolic Blood Pressure/100) ^3 | -0.032 | -0.054 | 10 |
| ln(respiratory rate/10) | 0.034 | 0.113 | 10 |
| Heart Rate | 0.003 | 0.073 | 10 |
| Male | 0.165 | 0.082 | 10 |
| No Hypertension | 0.109 | 0.049 | 10 |
| Not Pregnant | -0.435 | -0.035 | 10 |
| No Immunosuppression (HIV) | 0.059 | 0.02 | 9 |
| No Cough | 0.132 | 0.026 | 10 |
| No Other chronic lung disease | -0.366 | -0.018 | 10 |
| No Fever | 0.055 | 0.007 | 8 |
| Constant | -2.117 | -3.88 |  |
| **Lasso variable selection (unrestricted) (Continuous variables modelled using TEWS categories)** | | |  |
| C-statistic: 0.864 | | |  |
| CITL: -0.015 | | |  |
| **Parameter** | **Coefficient** | | **Number of times** |
|  | **Unstandardised** | **Standardised** |  |
| Age | 0.025 | 0.441 | 10 |
| No Supplemental Oxygen | -1.71 | -0.492 | 10 |
| Saturation (point increase TEWS) | 0.409 | 0.394 | 10 |
| No Diabetes | -0.57 | -0.222 | 10 |
| AVCPU (point increase TEWS) | 0.41 | 0.296 | 10 |
| Respiratory Rate (point increase TEWS) | 0.195 | 0.118 | 10 |
| No Hypertension | 0.134 | 0.06 | 10 |
| Heart Rate (point increase TEWS) | 0.076 | 0.070 | 10 |
| No Heart Disease | -0.102 | -0.04 | 10 |
| Not Pregnant | -0.473 | -0.038 | 10 |
| Male | 0.136 | 0.068 | 10 |
| No Immunosuppression (HIV) | 0.067 | 0.025 | 10 |
| No Cough | 0.111 | 0.023 | 10 |
| Temperature (point increase TEWS) | 0.1 | 0.057 | 10 |
| No Other chronic lung disease | -0.346 | -0.017 | 10 |
| Systolic Blood Pressure (point increase TEWS) | -0.032 | -0.014 | 10 |
| No Fever | 0.003 | 0.0003 | 9 |
| Constant | -3.005 | -3.888 |  |

S11 Figure: Calibration plots split internal validation complete case analysis

1. Unrestricted (continuous variables modelled fractional polynomials)


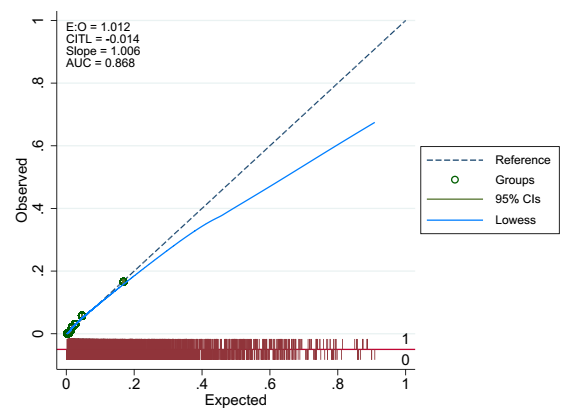


1. Restricted (continuous variables modelled fractional polynomials)


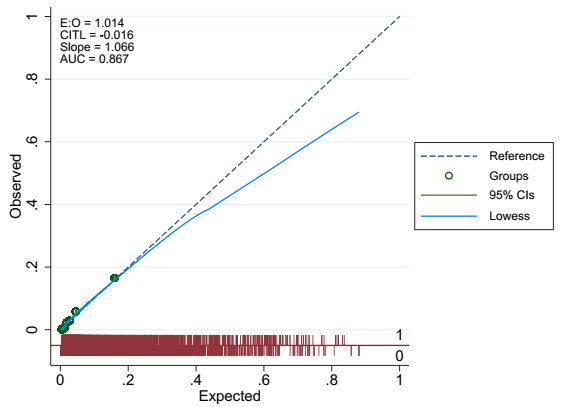


1. Unrestricted Lasso (continuous variables categorised)


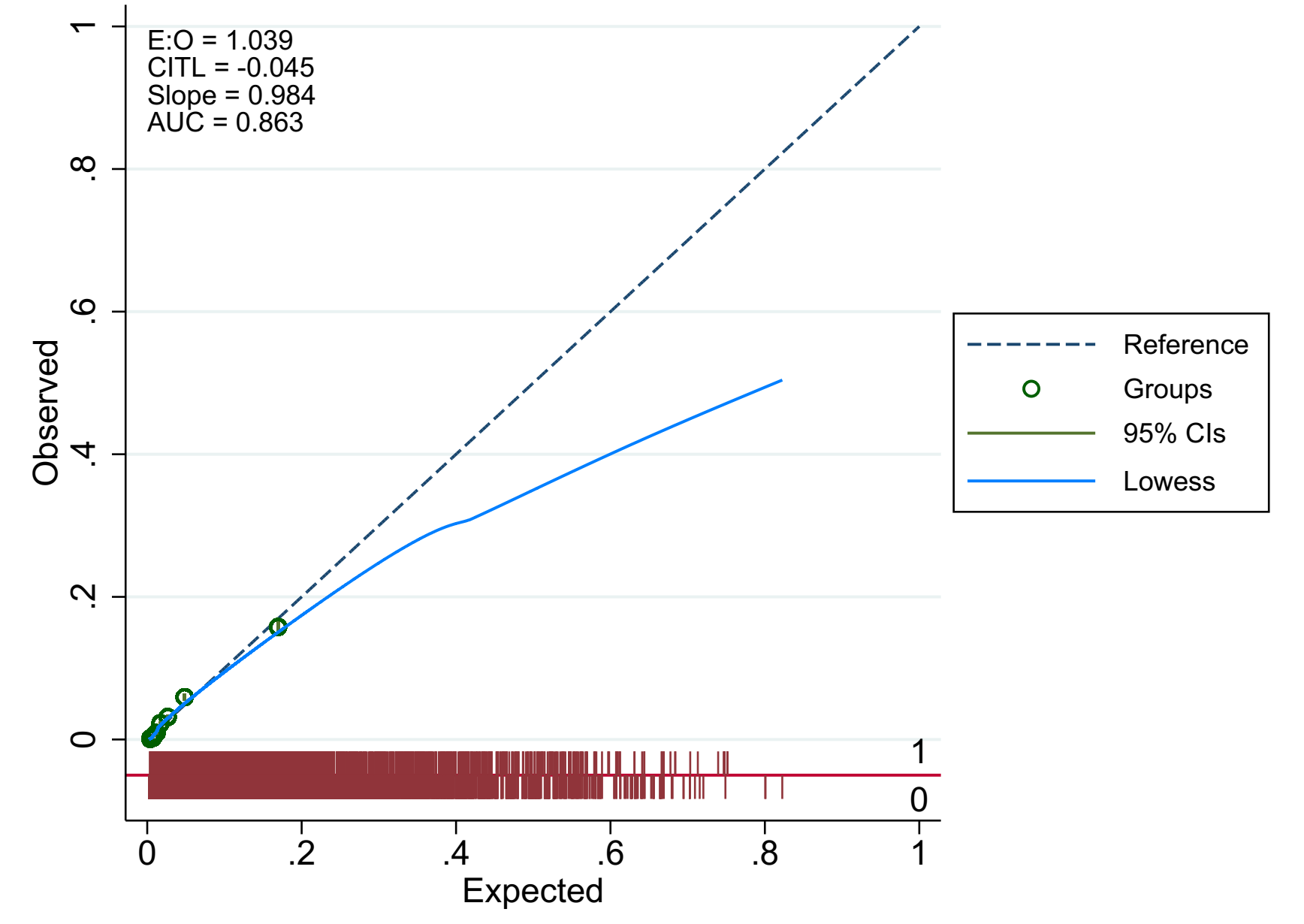


1. Restricted Lasso (continuous variables categorised)


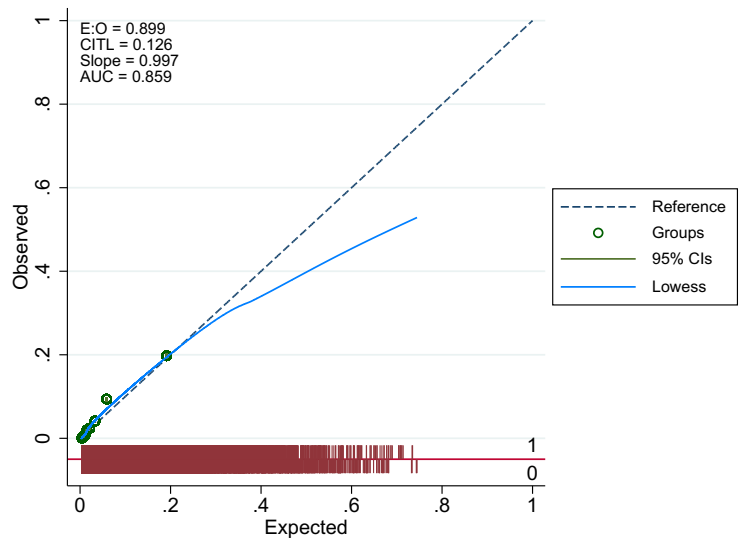


S12 Figure: Calibration plots split internal validation deterministic imputation

1. Unrestricted


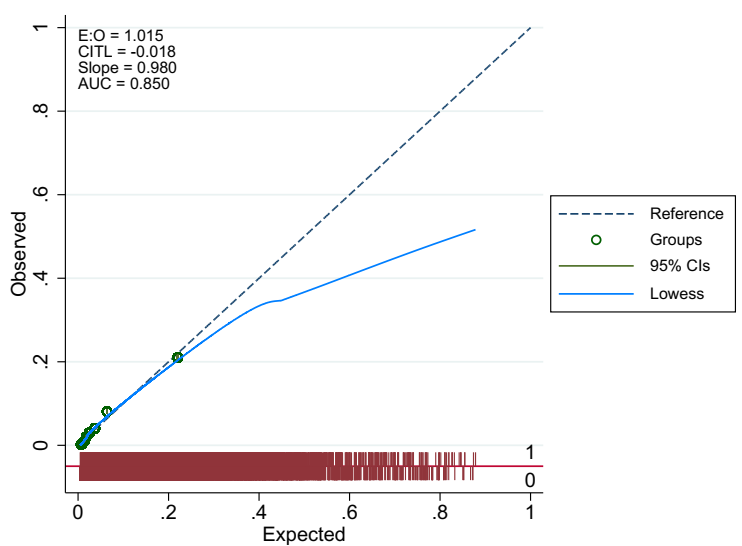


1. Restricted


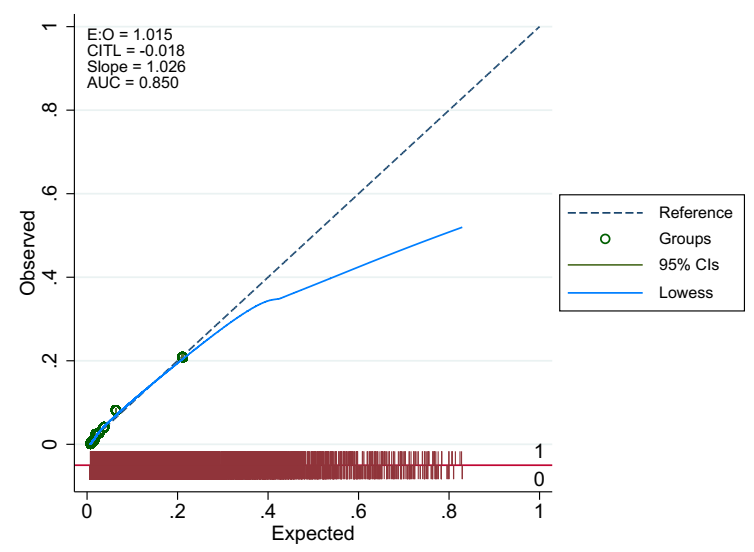


S13 Figure: ROC curves for predicting secondary outcome death for LMIC-PRIEST score

1. Development cohort (N=305,564) C stat 0.8346 (95% CI 0.83081 to 0.83835)

1. Omicron Validation cohort (N=140,520) C-stat 0. 0.8208 (95% CI: 0.81133 to 0.83025)

1. UK PRIEST Validation cohort (N=20,695) C-stat 0.7923 (95%CI: 0.78561 to 0.79903)

S14 Figure: ROC curves for predicting secondary outcome ICU admission/organ support for LMIC-PRIEST Score

1. Development cohort (N=305,564) C stat 0.7361 (95% CI 0.72403 to 0.74822)

1. Omicron Validation cohort (N=140,520) C-stat 0.6765 (95% CI: 0.65797 to 0.69506)

1. UK PRIEST validation cohort (20,965) C-stat 0.7019 (95% CI: 0.69157 to 0.71216)

S15 Table: Sensitivity, specificity, PPV, NPV and proportion with a positive score at each LMIC-PRIEST score threshold for predicting the primary outcome model development cohort (N=305,564)

|  | Proportion with score | Sensitivity | Specificity | NNP | PPV |
| --- | --- | --- | --- | --- | --- |
| >0 | 98.1% | 0.997 (0.996,0.998) | 0.02 (0.019,0.02) | 0.993 (0.99,0.995) | 0.042 (0.041,0.043) |
| >1 | 88.5% | 0.99 (0.989,0.992) | 0.119 (0.118,0.121) | 0.997 (0.996,0.997) | 0.046 (0.045,0.047) |
| >2 | 72.5% | 0.974 (0.972,0.977) | 0.286 (0.285,0.288) | 0.996 (0.996,0.997) | 0.056 (0.055,0.057) |
| >3 | 57.9% | 0.949 (0.945,0.952) | 0.437 (0.435,0.439) | 0.995 (0.995,0.995) | 0.068 (0.067,0.069) |
| >4 | 46.4% | 0.904 (0.899,0.91 | 0.555 (0.553,0.557) | 0.993 (0.992,0.993) | 0.081 (0.079,0.082) |
| >5 | 36.7% | 0.846 (0.84,0.853) | 0.654 (0.652,0.656) | 0.99 (0.99,0.99) | 0.095 (0.094,0.097) |
| >6 | 28.4% | 0.773 (0.765,0.780) | 0.737 (0.736,0.739) | 0.987 (0.986,0.987) | 0.112 (0.11,0.114) |
| >7 | 21.4% | 0.679 (0.671,0.687) | 0.806 (0.804,0.807) | 0.983 (0.983,0.984) | 0.131 (0.128,0.133) |
| >8 | 15.6% | 0.57 (0.561,0.578) | 0.862 (0.861,0.863) | 0.979 (0.978,0.98) | 0.151 (0.148,0.154) |
| >9 | 11% | 0.462 (0.453,0.470) | 0.905 (0.904,0.906) | 0.975 (0.974,0.976) | 0.173 (0.169, 0.177) |
| >10 | 7.3% | 0.353 (0.345,0.362) | 0.939 (0.938, 0.940) | 0.971 (0.971,0.972) | 0.199 (0.194,0.205) |
| >11 | 5.7% | 0.261 (0.254,0.269) | 0.962 (0.962,0.963) | 0.968 (0.967,0.969) | 0.23 (0.223,0.237) |
| >12 | 2.9% | 0.181 (0.174,0.187) | 0.978 (0.977,0.979) | 0.965 (0.965,0.966) | 0.261 (0.252,0.27) |
| >13 | 1.6% | 0.108 (0.103,0.114) | 0.988 (0.988, 0.988) | 0.963 (0.962, 0.963) | 0.281 (0.268, 0.294) |
| >14 | 0.9% | 0.067 (0.062, 0.071) | 0.994 (0.993, 0.994) | 0.961 (0.96, 0.962) | 0.309 (0.291, 0.327) |
| >15 | 0.5% | 0.036 (0.033, 0.04) | 0.997 (0.996,0.997) | 0.96 (0.959, 0.961) | 0.317 (0.293, 0.342) |
| >16 | 0.2% | 0.019 (0.017, 0.021) | 0.998 (0.998,0.999) | 0.959 (0.959, 0.960) | 0.336 (0.301,0.372 |
| >17 | 0.1% | 0.009(0.008, 0.011) | 0.999 (0.999, 0.999) | 0.959 (0.958, 0.96) | 0.326 (0.278, 0.377) |

S16 Table: Sensitivity, specificity, PPV, NPV and proportion with a positive score at each LMIC-PRIEST score threshold for predicting the primary outcome model Omicron validation cohort (N= N=140,520)

|  | Proportion with score | Sensitivity | Specificity | NNP | PPV |
| --- | --- | --- | --- | --- | --- |
| >0 | 97.9% | 0.998 (0.995,0.999) | 0.021 (0.02,0.022) | 0.998 (0.996,0.999) | 0.02 (0.02,0.021) |
| >1 | 87.7% | 0.995 (0.991,0.997) | 0.126 (0.124,0.128) | 0.999 (0.999,1) | 0.022 (0.022,0.023) |
| >2 | 70.7% | 0.962 (0.954,0.969) | 0.298 (0.295,0.3) | 0.997 (0.997,0.998) | 0.027 (0.026,0.028) |
| >3 | 55.3% | 0.929 (0.918,0.938) | 0.454 (0.452,0.457) | 0.997 (0.996,0.997) | 0.033 (0.032,0.035) |
| >4 | 43.1% | 0.841 (0.827,0.854) | 0.577 (0.575,0.58) | 0.994 (0.994,0.995) | 0.039 (0.037,0.04) |
| >5 | 32.9% | 0.766 (0.75,0.782) | 0.68 (0.677,0.682) | 0.993 (0.993,0.994) | 0.046 (0.044,0.048) |
| >6 | 24.6% | 0.663 (0.645,0.68) | 0.763 (0.761,0.765) | 0.991 (0.991,0.992) | 0.054 (0.051,0.056) |
| >7 | 17.6% | 0.555 (0.537,0.574) | 0.831 (0.829,0.833) | 0.989 (0.989,0.99) | 0.063 (0.06,0.066) |
| >8 | 12% | 0.443 (0.425,0.462) | 0.886 (0.885,0.888) | 0.987 (0.987,0.988) | 0.073 (0.069,0.077) |
| >9 | 11% | 0.339 (0.321,0.357) | 0.928 (0.926,0.929) | 0.986 (0.985,0.986) | 0.087 (0.081, 0.092) |
| >10 | 4.9% | 0.238 (0.222,0.254) | 0.955 (0.954, 0.957) | 0.984 (0.983,0.985) | 0.098 (0.091,0.105) |
| >11 | 2.9% | 0.159 (0.146,0.173) | 0.974 (0.973,0.975) | 0.983 (0.982,0.984) | 0.111 (0.101,0.121) |
| >12 | 1.7% | 0.115 (0.103,0.127) | 0.985 (0.984,0.985) | 0.982 (0.981,0.983) | 0.132 (0.119,0.146) |
| >13 | 1% | 0.076 (0.067, 0.087) | 0.991 (0.991, 0.992) | 0.981 (0.981, 0.982) | 0.148 (0.13, 0.168) |
| >14 | 0.5% | 0.044 (0.037, 0.052) | 0.995 (0.995, 0.996) | 0.981 (0.98, 0.982) | 0.162 (0.136, 0.19) |
| >15 | 0.3% | 0.027 (0.022, 0.034) | 0.997 (0.997,0.998) | 0.981 (0.98, 0.981) | 0.166 (0.133, 0.204) |
| >16 | 0.2% | 0.016 (0.012, 0.022) | 0.998 (0.998,0.999) | 0.98 (0.98, 0.981) | 0.179 (0.133,0.232) |
| >17 | 0.1% | 0.006 (0.004, 0.01) | 0.999 (0.999, 0.999) | 0.98 (0.98, 0.98.1) | 0.157 (0.095, 0.24) |

S17 Table: Sensitivity, specificity, PPV, NPV and proportion with a positive score at each LMIC-PRIEST score threshold for predicting the primary outcome model UK PRIEST validation cohort ( N= N=20,698)

|  | Proportion with score | Sensitivity | Specificity | NNP | PPV |
| --- | --- | --- | --- | --- | --- |
| >0 | 99.4% | 1 (0.998,1) | 0.007 (0.006,0.008) | 0.983 (0.94,0.998) | 0.22 (0.22,0.23) |
| >1 | 94.7% | 0.999 (0.997,1) | 0.067 (0.063,0.071) | 0.994 (0.988,0.998) | 0.233 (0.227,0.239) |
| >2 | 88.4% | 0.996 (0.993,0.997) | 0.147 (0.142,0.153) | 0.992 (0.987,0.995) | 0.147 (0.142,0.153) |
| >3 | 81.4% | 0.988 (0.984,0.991) | 0.235 (0.229,0.242) | 0.985 (0.981,0.989) | 0.268 (0.262,0.275) |
| >4 | 73.7% | 0.971 (0.966,0.976) | 0.329 (0.322,0.337) | 0.976 (0.971,0.98 | 0.292 (0.284,0.299) |
| >5 | 65.2% | 0.942 (0.935,0.949) | 0.43 (0.422,0.437) | 0.963 (0.958,0.967) | 0.319 (0.311,0.327) |
| >6 | 56.5% | 0.891 (0.881,0.90) | 0.527 (0.52,0.525) | 0.944 (0.94,0.949) | 0.349 (0.34,0.357) |
| >7 | 47.1% | 0.818 (0.806,0.829) | 0.628 (0.62,0.635) | 0.924 (0.919,0.929) | 0.384 (0.375,0.394) |
| >8 | 37.7% | 0.713 (0.70,0.727) | 0.718 (0.711,0.725) | 0.898 (0.893,0.903) | 0.418 (0.407,0.43) |
| >9 | 28.9% | 0.599 (0.584,0.613) | 0.798 (0.792,0.804) | 0.875 (0.87,0.88) | 0.457 (0.445, 0.47) |
| >10 | 21% | 0.466 (0.452,0.481) | 0.863 (0.857, 0.868) | 0.851 (0.845,0.856) | 0.491 (0.476,0.506) |
| >11 | 14.6% | 0.351 (0.337,0.365) | 0.912 (0.908,0.917) | 0.832 (0.826,0.837) | 0.533 (0.515,0.551) |
| >12 | 9.7% | 0.252 (0.239,0.264) | 0.947 (0.944,0.951) | 0.817 (0.811,0.822) | 0.575 (0.553,0.597) |
| >13 | 6% | 0.169 (0.158, 0.18) | 0.971 (0.968, 0.973) | 0.804 (0.799, 0.81) | 0.621 (0.594, 0.648) |
| >14 | 3.6% | 0.105 (0.965, 0.115) | 0.984 (0.982, 0.986) | 0.795 (0.789, 0.80) | 0.655 (0.619, 0.689) |
| >15 | 1.9% | 0.06 (0.054, 0.068) | 0.992 (0.991,0.994) | 0.788 (0.782, 0.794) | 0.695 (0.647, 0.74) |
| >16 | 1% | 0.032 (0.027, 0.038) | 0.997 (0.996,0.998) | 0.784 (0.778, 0.789) | 0.739 (0.672,0.798) |
| >17 | 0.4% | 0.017 (0.013, 0.021) | 0.999 (0.998, 0.999) | 0.781 (0.776, 0.787) | 0.826 (0.733, 0.897) |

.

S18 Chart:  Probability of primary adverse outcome for each value of the LMIC-PRIEST score

1. Development cohort (N=305,564)
2. Omicron Validation cohort
3. UK PRIEST validation cohort
